# Increasing Consumer Engagement by Tailoring a Public Reporting Website on the Quality of Diabetes Care: A Qualitative Study

**DOI:** 10.2196/jmir.6555

**Published:** 2016-12-21

**Authors:** Maureen A Smith, Lauren Bednarz, Peter A Nordby, Jennifer Fink, Robert T Greenlee, Daniel Bolt, Elizabeth M Magnan

**Affiliations:** ^1^ Health Innovation Program School of Medicine and Public Health–Madison University of Wisconsin Madison, WI United States; ^2^ Department of Population Health Sciences School of Medicine and Public Health–Madison University of Wisconsin Madison, WI United States; ^3^ Department of Family Medicine and Community Health University of Wisconsin School of Medicine and Public Health–Madison Madison, WI United States; ^4^ Department of Health Informatics College of Health Sciences University of Wisconsin–Milwaukee Milwaukee, WI United States; ^5^ Aurora Research Institute Aurora Health Care Milwaukee, WI United States; ^6^ Center for Clinical Epidemiology Marshfield Clinic Research Foundation Marshfield, WI United States; ^7^ Department of Educational Psychology University of Wisconsin–Madison Madison, WI United States; ^8^ Department of Family and Community Medicine University of California, Davis Sacramento, CA United States

**Keywords:** diabetes, chronic conditions, public reports, patient engagement

## Abstract

**Background:**

The majority of health care utilization decisions in the United States are made by persons with multiple chronic conditions. Existing public reports of health system quality do not distinguish care for these persons and are often not used by the consumers they aim to reach.

**Objective:**

Our goal was to determine if tailoring quality reports to persons with diabetes mellitus and co-occurring chronic conditions would increase user engagement with a website that publicly reports the quality of diabetes care.

**Methods:**

We adapted an existing consumer-focused public reporting website using adult learning theory to display diabetes quality reports tailored to the user’s chronic condition profile. We conducted in-depth cognitive interviews with 20 individuals who either had diabetes and/or cared for someone with diabetes to assess the website. Interviews were audiotaped and transcribed, then analyzed using thematic content analysis.

**Results:**

Three themes emerged that suggested increased engagement from tailoring the site to a user’s chronic conditions: ability to interact, relevance, and feeling empowered to act.

**Conclusions:**

We conclude that tailoring can be used to improve public reporting sites for individuals with chronic conditions, ultimately allowing consumers to make more informed health care decisions.

## Introduction

In spite of evidence that consumers want more information on health care provider performance, there is limited use of current reports to make informed health care decisions [1,2] because only 12% of US adults have consulted online rankings or reviews of clinicians or other physicians [3]. Public reporting on health care performance has been available in the United States for more than two decades and was further enhanced with the Affordable Care Act’s [4] creation of a “national strategy for quality improvement through publicly reporting quality performance” [5]. Consequently, health care information transparency initiatives are rapidly increasing. For example, the recently formed Center for Healthcare Transparency has a goal of making information on the relative cost and quality of health care services available to 50% of the US population by 2020 [6]. Yet, studies suggest that consumers do not seek out this information, understand it, trust it, or know how to use it [7,8]. Promising strategies to increase consumer engagement in public reports are to improve the design [9,10], include patient narrative [11-14], and to tailor metrics to reflect the concerns and preferences of individual consumers [15]. It has been shown that the absence of tailoring in public reports makes them unlikely to succeed [16,17] and that consumers are deterred by the content and design of current reports that lack tailoring to their individual needs [18]. Tailoring, or personalizing health information, means creating communicative information about a given individual to determine what specific content they will receive, the contexts or frames surrounding the content, by whom it will be presented, and the channels through which it will be delivered [19].

Consumers with multiple chronic conditions are a priority population for tailoring public reports because they have a continuing need to know how to best manage their health conditions to avoid complications and improve their health [20]. It is increasingly recognized that existing reporting initiatives do not support decision making for these consumers even though persons with chronic conditions may be the population most interested in public reports [7,21]. Those with diabetes mellitus are particularly representative of this group because more than 90% have multiple chronic conditions (diabetes plus at least one more condition) [22]. Additionally, persons with diabetes may be more receptive to publicly reported information on quality [23] because of their emotional connection to their disease, awareness of symptoms and consequences, and information-seeking behaviors.

We examined whether tailoring existing public reports on health care quality to persons with diabetes and co-occurring chronic conditions would increase their engagement with the reports. Using four principles of adult learning [24], we adapted a consumer-focused website that publicly reports the quality of diabetes care using a hypothetical patient narrative [25] by tailoring the information presented more specifically to each person’s co-occurring chronic conditions. We shared the original and revised websites with consumers and conducted semistructured interviews to obtain feedback on the changes. We expected that greater customization of content tailored to the individual’s conditions would increase engagement with this public reporting website [26].

## Methods

### Study Design

As recommended in the 2025 vision for public reporting [20], we engaged consumers in developing and testing public reports as a mechanism to determine what is most useful and meaningful to them. We adapted an existing public reporting website that uses a novel storytelling format to explore the health issues and health care options of “Helen,” who has diabetes [27]. In its existing format, consumers can follow and learn from Helen’s story, compare the performance of more than 20 health systems on metrics representing the quality of diabetes care within each health system, and find useful tips about being a better health care consumer. For our study, we redesigned the website to tell the story of “Karen,” who has diabetes and other health issues (Multimedia Appendix 1 Figure A1). The website redesign was interactive and allowed the content to be tailored to each user to reflect health system performance metrics for individuals with similar chronic conditions. Next, to assess the websites, we conducted in-depth cognitive interviews with 20 individuals who either had diabetes and/or cared for someone with diabetes [28]. During the interviews, we asked participants to navigate through the existing website with Helen’s story and the redesigned website with Karen’s story. Interviews were audiotaped and transcribed, then analyzed using thematic content analysis.

### Developing a Website That Tailors Information to the User

To create a website that tailors information for the user, we used four principles of adult learning: (1) adult learners’ experience, (2) involved adult learner, (3) problem-centered, and (4) relevance and impact to learners’ lives [24]. Our adaptations to address these principles included (1) creating a new story about Karen who has other health issues in addition to diabetes, (2) allowing the user to answer four questions about their own chronic conditions, (3) using the four questions to create a chronic condition profile for the user (Multimedia Appendix 1 Figure A2), and (4) generating a report comparing quality among health systems for that user’s chronic condition profile.

### Sampling

To recruit, flyers advertising the study were posted at local libraries, grocery stores, community centers, senior centers, university buildings, and a local hospital. Additionally, we placed an ad in a local paper. Only individuals who had diabetes and/or cared for an individual with diabetes were eligible. Those interested in participating in the study were screened via telephone by the University of Wisconsin Survey Center. We expected our study population to be older, white, and college educated based on the demographics of the local population. Once we reached 20 participants, we closed enrollment into the study. We based our sample size on the goal of saturation (ie, little new is being learned) [29,30]. Each participant received a US $50 stipend on the completion of their interview.

### Data Collection

Between April and May 2015, we conducted 20 one-on-one, hour-long, in-depth cognitive interviews. During the interviews, the participants navigated through the existing consumer-focused Web pages with Helen’s story while being asked scripted, open-ended questions related to (1) navigation of content, (2) validity of the content, and (3) understanding of the displays. The participants were then instructed to navigate through the newly adapted Web pages with Karen’s story while being asked the same scripted, open-ended questions plus questions about (4) their understanding of information presented for the chosen chronic condition profile, and (5) perceived value of the health care quality report presented for the user’s chronic condition profile. The interviews were audio recorded and transcribed. Short summary thoughts and notes were drafted after each interview.

### Data Analysis

We used thematic content analysis to identify, analyze, and report themes within our data. As a guide, we applied Braun and Clarke’s “six phases of analysis” in which coders (1) familiarize themselves with the data, (2) generate codes, (3) search for themes among codes, (4) review themes, (5) define and name themes, and (6) produce the report [31]. Data analysis was performed by two research assistants who independently coded each transcript in full and met regularly to discuss coding and reach interrater reliability. Agreed-on codes were then examined to identify reoccurring themes in our dataset.

## Results

Our study population (n=20) was primarily female (n=13) with racial minorities making up 30% (6/20) of our sample (Table 1). The median age range of our participants was between 41 and 50 years old. The majority (60%, 12/20) of participants had health insurance either through Medicare (n=4), Medicaid (n=8), or a mix of Medicaid and/or Medicare supplemented with private insurance (n=5). Participants included 8 persons with diabetes, 8 caregivers of persons with diabetes, and 4 participants who were both persons with diabetes and caregivers for persons with diabetes.

### Themes

The findings from our thematic content analysis support the concept that tailoring a public reporting website to a user’s chronic condition profile can increase consumer engagement. Participants found that the ability to interact (Theme 1) with the website to generate a chronic condition profile personalized their experience, whereas the addition of Karen’s story to the website increased the relevance (Theme 2) of the site because it presented the voice of an individual who has “other health issues” in addition to diabetes (Textbox 1). The presence of these two themes—ability to interact and relevance—led to participants’ feeling empowered to act (Theme 3) to improve their health care and health.

**Themes identified and relevant quotes from interviews with consumers’ while navigating a public reporting website on health care quality tailored to a user’s chronic condition profile compared to a nontailored website.**

**Theme 1: Ability to interact**

- “It’s nice that, like, you can kind of put your conditions in and see how people are performing.”
- “Well, right off the bat this is more personal because it’s involving my opinion or self-data. So, it’s more personal.”
- “They should maybe have a box where you could actually type in comments because you might want to specify what issues you’re dealing with. I would like to provide more information.”

**Theme 2: Relevance**

- “...it’s really good to hear personal stories of people going through the same things—just to be able to relate to that...”
- “She [Karen] said that she had other health problems in addition to diabetes, which a lot of people have and can probably relate to.”
- “This one’s [Karen’s] a lot better. I just realized that this one [Helen’s] tells you nothing, nothing at all. This one right here is pretty much breaking it down for me. Karen’s—I can relate to her more.”

**Theme 3: Feeling empowered to act**

- “I think that it’s just a way of knowing which health system would take care of you the best to manage your disease.”
- “Well, if I’m changing insurance, and I have the ability to choose a doctor, I want to choose a doctor that knows what they’re doing.”
- “It’s important to do your research, because that is true, some doctors, hospitals, and clinics are better when they’re dealing with patients with multiple issues...in addition to diabetes. I didn’t know that.”

**Table 1:** Participant characteristics (n=20).

Participant characteristics		n (%)
**Age range (years)**		
	18-30	5 (25)
	31-40	2 (10)
	41-50	6 (30)
	51-60	6 (30)
	61-70	1 (5)
**Sex**		
	Female	13 (65)
	Male	7 (35)
**Race**		
	White	13 (65)
	Black or African American	5 (25)
	Other	1 (5)
	Unidentified	1 (5)
**Income (US $)**		
	<$25,000	8 (40)
	$25,000-$40,000	4 (20)
	$40,000-$65,000	6 (30)
	$75,000-$100,000	1 (5)
	Unknown	1 (5)
**Health insurance status**		
	Private	8 (40)
	Medicare	3 (15)
	Medicaid	4 (20)
	Mix of Medicaid and/or Medicare with Private	5 (25)
**Employment status**		
	Employed	13 (65)
	Unemployed	7 (35)
**Highest level of education**		
	High school diploma or equivalent	3 (15)
	Some college	7 (35)
	Associate’s degree	2 (10)
	Bachelor’s degree	7 (35)
	Master’s degree	1 (5)

### Ability to Interact

The process of answering questions about their chronic conditions allowed participants to interact directly with the site to create their chronic condition profile and, based on this profile, display information on which health systems perform best for a “patient like them.”

Participants saw value in interacting with the site to create a profile based on their conditions. One participant stated that it was nice to be able to select your conditions and receive more “personal” data. In fact, some participants would have liked the ability to interact with the site on an even more personal and comprehensive level via a comment box where they could be more specific about their health and conditions. Another participant suggested having a checklist of all possible conditions for the user to self-select. Our findings suggest that the more a user is allowed to interact on a website that reports on health care quality, the more engaged they may become with website and ultimately the information that the website provides.

Overwhelmingly, participants in the study found the website easy to navigate (90%, 18/20). Because the primary goal of the website was for participants to be able to create a tailored report on health care quality by self-selecting their conditions to create a chronic condition profile, the ease of navigating the website was an integral piece of allowing that interaction to take place.

### Relevance

Karen’s story (multiple chronic conditions in addition to diabetes) was viewed by the participants as being more relevant because Karen faced challenges similar to their own. Adding Karen’s story to the website provided a relatable narrator for the participants in our study. It was easy for them to “see themselves” in Karen’s story because she, like them, lives with diabetes and other chronic conditions. As one participant remarked, “There’s more to the issue of complicated diabetes...I don’t know many diabetics that don’t have other health issues.”

Participants viewed receiving a score on how health systems perform for an individual with diabetes and other health issues similar to theirs, as opposed to a score for all patients with diabetes, as significant to them as an individual. Providing a platform for consumers to identify their conditions, to create a profile, and receive a score on health system performance that is personalized for them allows consumers to “see” themselves in the data. This increased the relevance of the health care quality report being communicated because it was specifically related to the consumer and their chronic condition profile. When asked if the website tailored to their chronic condition profile provided more value compared to the untailored website, 13 of 20 participants said yes, 3 participants did not comment, and 4 found no additional value.

### Feeling Empowered to Act

The process of self-selecting their conditions and receiving personalized data on how health systems perform for a “patient like them” created a sense of empowerment for the participants. This feeling of empowerment was stimulated by providing a report that showed which health systems perform best in managing patients with their similar chronic condition profile.

Participants suggested that knowing how health systems perform for individuals similar to themselves may influence where they would seek out care. For example, one participant stated they would be more likely to see a provider if the data showed they were good at treating patients with a similar profile to theirs. Participants also thought they would find this information particularly valuable if they were changing insurance. This highlights the importance of making public reports available to consumers at times when they are most likely to make a decision about their health care including health care insurance coverage (eg, open enrollment periods).

Lastly, Karen’s narrative served as a prescriptive guide for participants on how they should interact with their provider to better manage their health. One participant stated, “If you don’t understand something, you have to be honest. And you have to say (to your provider), you know, ‘I don’t understand.’” Other participants felt empowered by the narrative to initiate conversations with their provider and become more of an active participant in their health.

## Discussion

The development of personalized or customizable report cards has been identified as an important priority to the success of consumer-focused quality reporting [32], although there has been a dearth of evidence on the consumer perspective on personalization [18]. By engaging consumers, we found that tailoring a website that publicly reports on health care quality to a consumer’s chronic condition profile increased consumer engagement with the website in specific ways. The themes that emerged from our analysis suggest that consumers value a public reporting website that they can interact with, that they feel is relevant to their situation, and that provides them with a feeling of empowerment to support action. Our findings support that tailoring is indeed a potential strategy for increasing consumer engagement in public reports as suggested by Huckman [15].

Our specific approach of tailoring a public reporting website based on the principles of adult learning as a framework represents a useful guide to increase consumer engagement. Multiple different strategies have been proposed to increase consumer engagement in public reporting, such as providing cost data, having patient comments and stories on the sites, better design, and providing information that is more relevant to the user [18,33]. However, there has been a lack of an overarching framework to organize and prioritize these multiple strategies. Our framework is consistent with consumer requests to have public reporting websites provide more personally relevant information on health care quality for patients with similar health conditions [34]. Also supporting our framework, we found that the participants in our study wish to engage further with the website we adapted. For example, participants expressed a desire to enter more detailed information about their health conditions in order to create an even more personalized quality report. This suggests that ensuring the delivery of public reports on health care quality are adequately personalized to the consumer is important to consider because personalization is an important component of effectiveness [35,36]. In other settings, tailoring educational materials to individual consumers increases the chances that the information will be read and remembered, saved and discussed with others, perceived as interesting, and personally relevant [37].

There are multiple ways to tailor health information, such as matching to race, ethnicity, gender, or age [34], but we chose to tailor reports for persons with multiple chronic conditions for a variety of reasons. Currently in the United States, the majority of health care utilization decisions are made by the quarter of individuals who have multiple chronic conditions, comprising approximately 66% of health care expenditures in the United States [38]. Having chronic conditions is a powerful motivator to seek out the best health care available because individuals with chronic conditions have a continuing need to know how to best manage their health conditions to avoid complications or deterioration, minimize symptoms, and improve their health [21]. In addition, individuals with chronic conditions see multiple providers of potentially differing quality in the face of complex medical needs [21]. By allowing consumers to tailor the reports to their chronic condition profile, we are delivering content that is relevant to their interests and needs [26].

Our study has several limitations. First, although building on the design of a preexisting consumer website as a basis for our assessment reduced development time and facilitated real-world testing, it constrained our options in presentation of data. Second, we had an overrepresentation of low-income and racial minorities in our sample compared to the expected population. However, our sample represents a high-priority population as a proposed strategy to reduce health disparities and improve health outcomes by making health information, such as public reports available to racial and ethnic minorities [39].

Although consumers want more transparency and information on health care provider performance, they do not use existing public websites that provide this information. We found that redesigning a public reporting website using adult learning theory is one way to potentially increase consumer engagement with these sites. Participants in our study valued their ability to interact with the website, felt the information on the site had relevance to them, and felt empowered to act based on the information provided. Our approach provides specific guidance on changing the content and format of public reports to engage and inform consumer decisions. A potential area for future study is how to tailor health system performance metrics to additional consumer attributes that might affect the quality of care (eg, race/ethnicity), to the relevance of diabetes in a person’s perception of health, or to awareness of other comorbidities, and how these might affect diabetes care.

## Appendix

Multimedia Appendix 1 Public reporting website storyboards.
