# Supplementary material for: Increasing Consumer Engagement by Tailoring a Public Reporting Website on the Quality of Diabetes Care: A Qualitative Study
Source: J Med Internet Res. 2016 Dec 21;18(12):e332. doi: 10.2196/jmir.6555 (PMC5214669; doi:10.2196/jmir.6555)

Appendix A

For our study, we adapted an existing public reporting website that uses a novel storytelling format to explore the health issues and healthcare options of “Helen,” who has diabetes (http://www.myhealthwi.org/Resources/GettingGoodCare.aspx). In its existing format, consumers can follow and learn from “Helen’s” story, compare the performance of over 30 health systems, and find useful tips about being a better healthcare consumer.

This Appendix describes how we redesigned the website to tell the story of “Karen” who has other health issues in addition to diabetes that influence the care she receives. The storyboard below visualizes the website that introduces Karen and includes allowing the user to answer four questions about their own chronic conditions while maintaining privacy about the exact conditions (Appendix 1a). The four questions were created by combining conditions into four groups based on previous research; within each group, the conditions had similar relationships to diabetes testing and control metrics.[14] Each user responded as to whether they had 0, 1, or 2+ conditions from the list of conditions in each of the four condition group. The website then generated an individual profile of the user’s chronic conditions. Each user was identified as one of 14 chronic condition profiles. Finally, the website generates a report comparing quality of care among health systems for that user’s chronic condition profile (Appendix 1b).

Appendix Figure A1

Storyboard for “Karen” and four questions to identify the user’s chronic condition profile

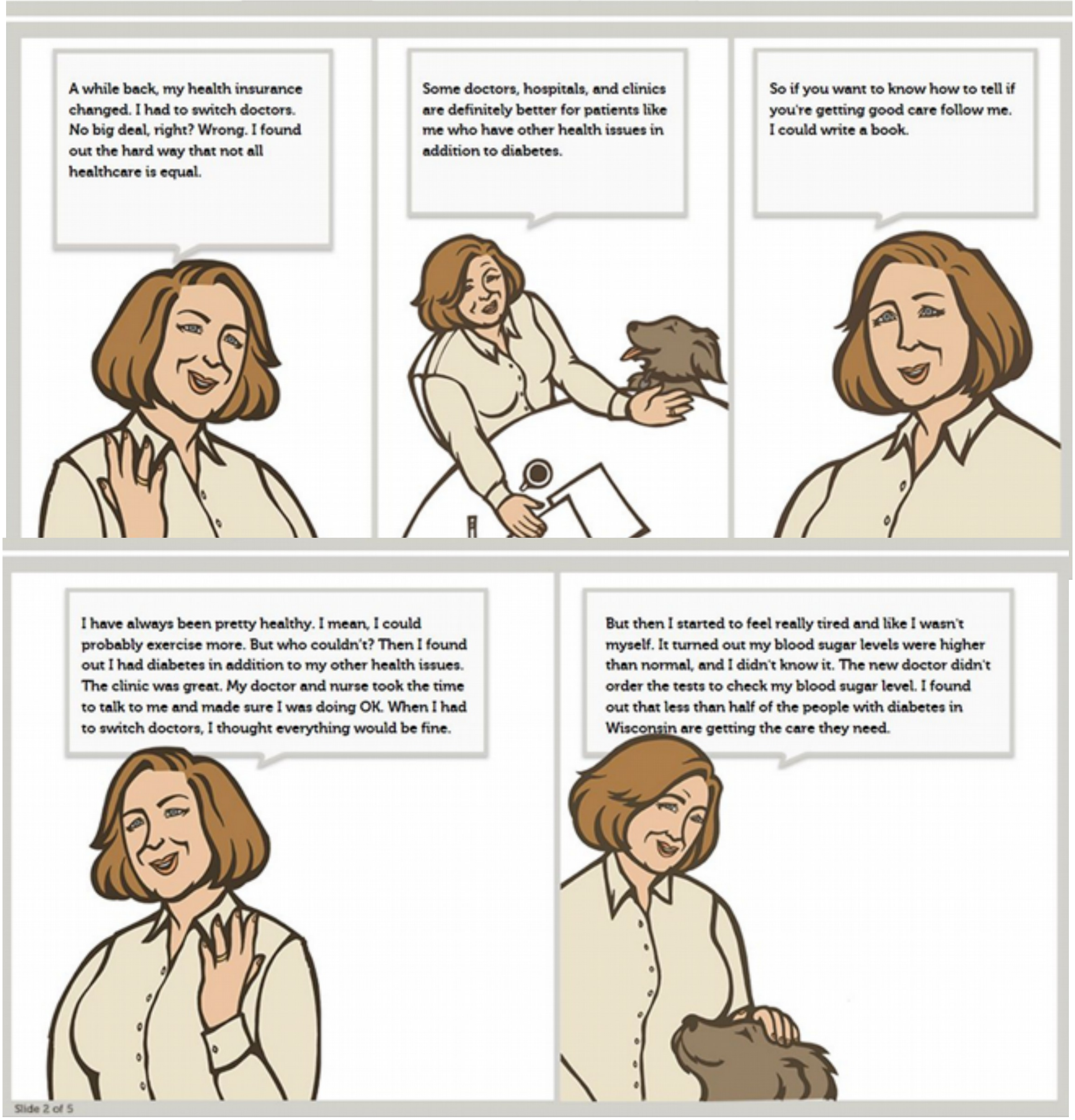

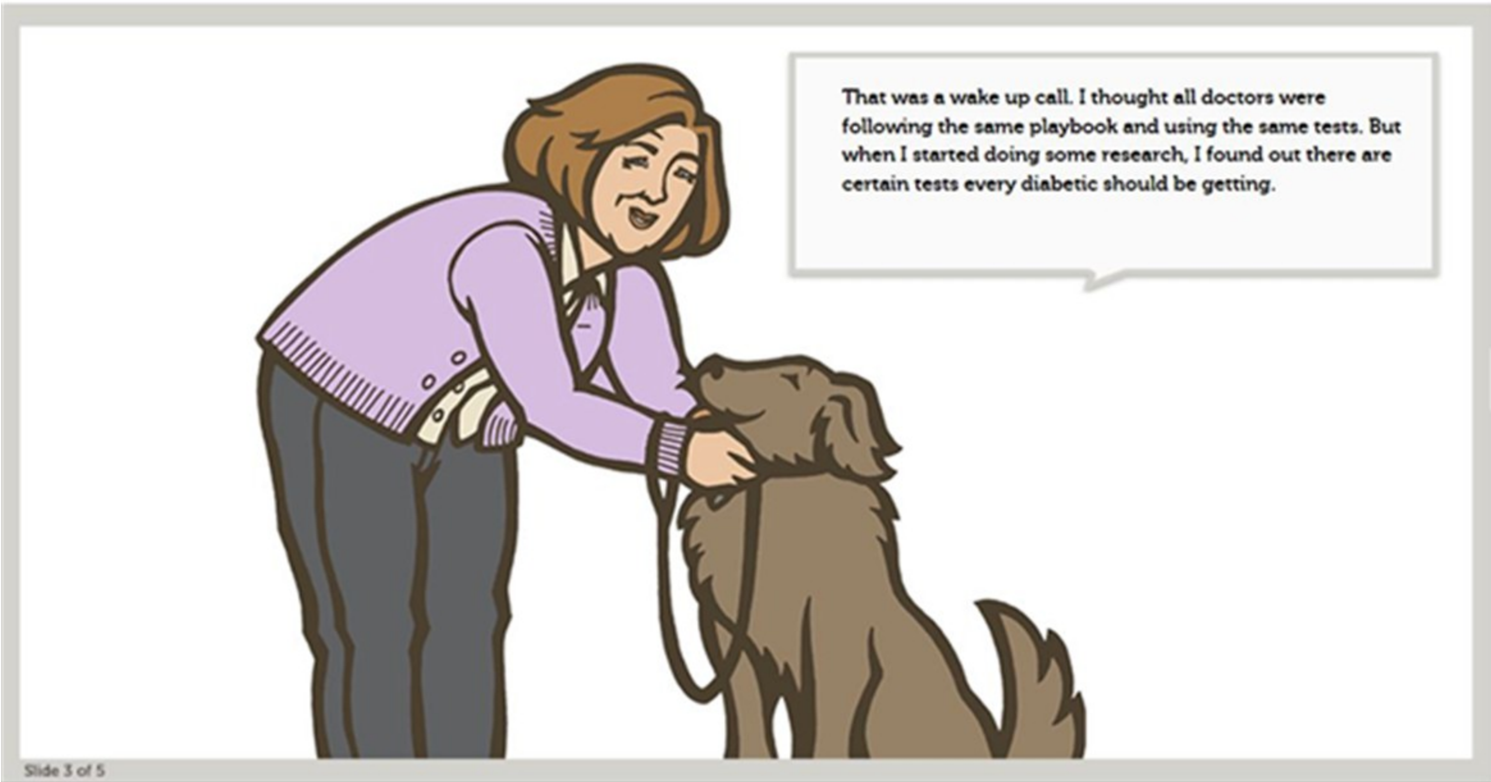
That was a wake up call. I thought all doctors were following the same playbook and using the same tests. But when I started doing some research, I found out there are certain tests every diabetic should be getting.
Slide 3 of 5

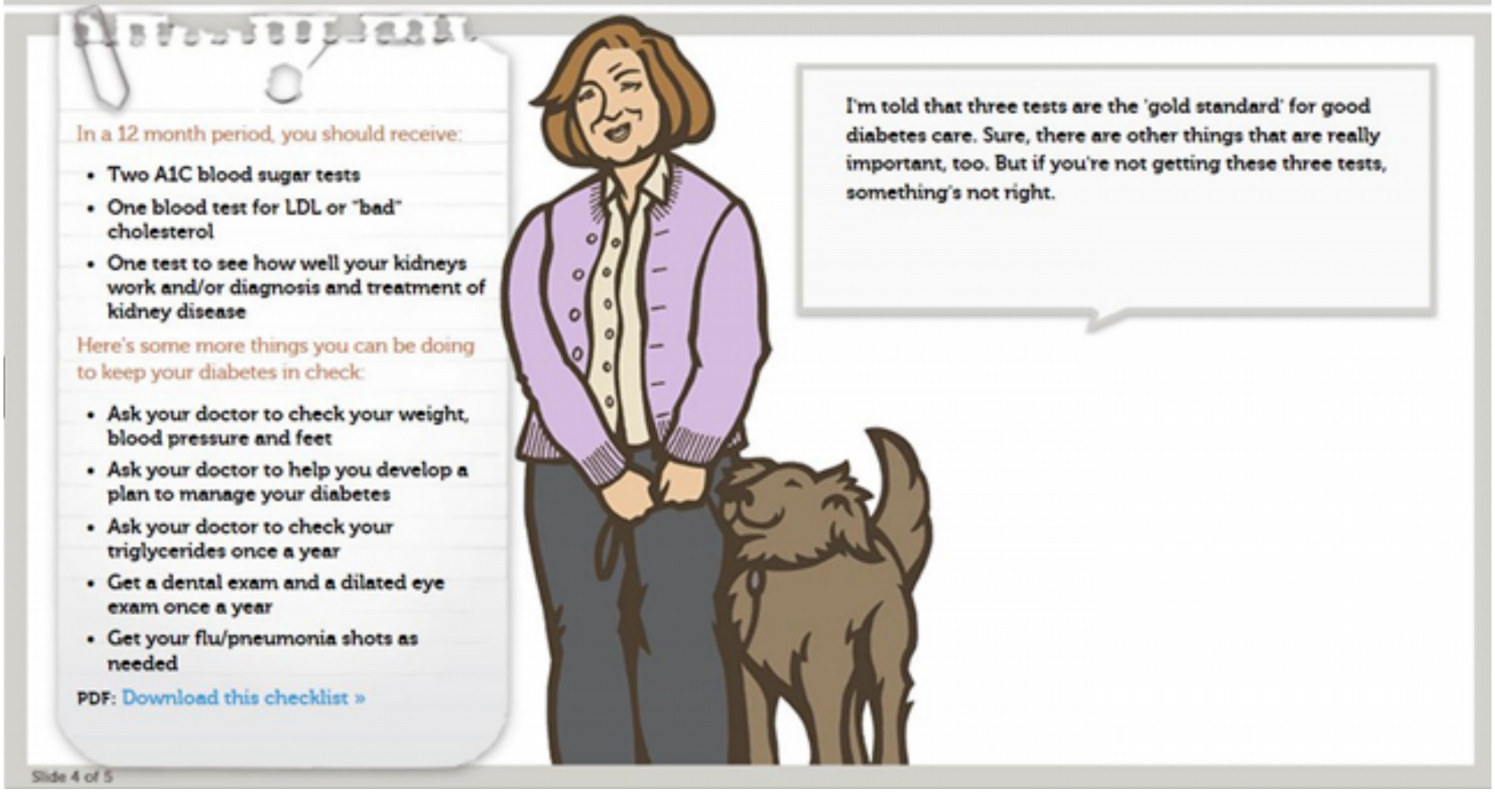
In a 12 month period, you should receive:
• Two A1C blood sugar tests
• One blood test for LDL or "bad" cholesterol
• One test to see how well your kidneys work and/or diagnosis and treatment of kidney disease
Here's some more things you can be doing to keep your diabetes in check:
• Ask your doctor to check your weight, blood pressure and feet
• Ask your doctor to help you develop a plan to manage your diabetes
• Ask your doctor to check your triglycerides once a year
• Get a dental exam and a dilated eye exam once a year
• Get your flu/pneumonia shots as needed
PDF: Download this checklist »
I'm told that three tests are the 'gold standard' for good diabetes care. Sure, there are other things that are really important, too. But if you're not getting these three tests, something's not right.
Slide 4 of 5

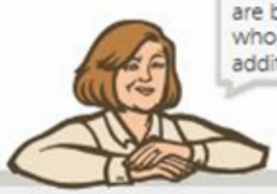

I found out that some health systems are better at treating patients like me who have other health issues in addition to diabetes.

Stories | Compare Scores | Tools

Karen's Diabetes

Please answer the following four questions to create a chart that shows the percent of patients like you getting the care they need.

1. How many of the following health issues do you have?

- Blockage in your heart arteries ("coronary heart disease", "hardening of the arteries of the heart")
- Cancer, any type
- High cholesterol
- Men: Enlarged prostate
- Women: Menopause or perimenopause symptoms

○ I have **none** of these health issues
○ I have **one** of these health issues
○ I have **two of more** of these health issues

2. How many of the following health issues do you have?

- Chronic liver disease
- Health issue you were born with not related to your heart
- High blood pressure
- Kidney disease (but not kidney failure)
- Men: disease of bladder, urinary tract, or reproductive organs (but not prostate enlargement)

○ I have **none** of these health issues
○ I have **one** of these health issues
○ I have **two of more** of these health issues

Health System #1

Health System #2

Health System #3

3. How many of the following health issues do you have?

- Congestive heart failure
- Disease of blood vessels not caused by blood clots or hardening of the arteries
- Migraines
- Problem with alcohol or drug use
- Women: disease of bladder, urinary tract, or reproductive organs (but not menopause)

○ I have **none** of these health issues
○ I have **one** of these health issues
○ I have **two of more** of these health issues

4. How many of the following health issues do you have?

- Disease of thyroid
- Kidney failure (with or without dialysis)
- Obesity
- Osteoarthritis
- Sleep disorders

○ I have **none** of these health issues
○ I have **one** of these health issues
○ I have **two of more** of these health issues

Select a measure

Appendix Figure A2

Website display of quality of care for state health systems for the user's chronic condition profile

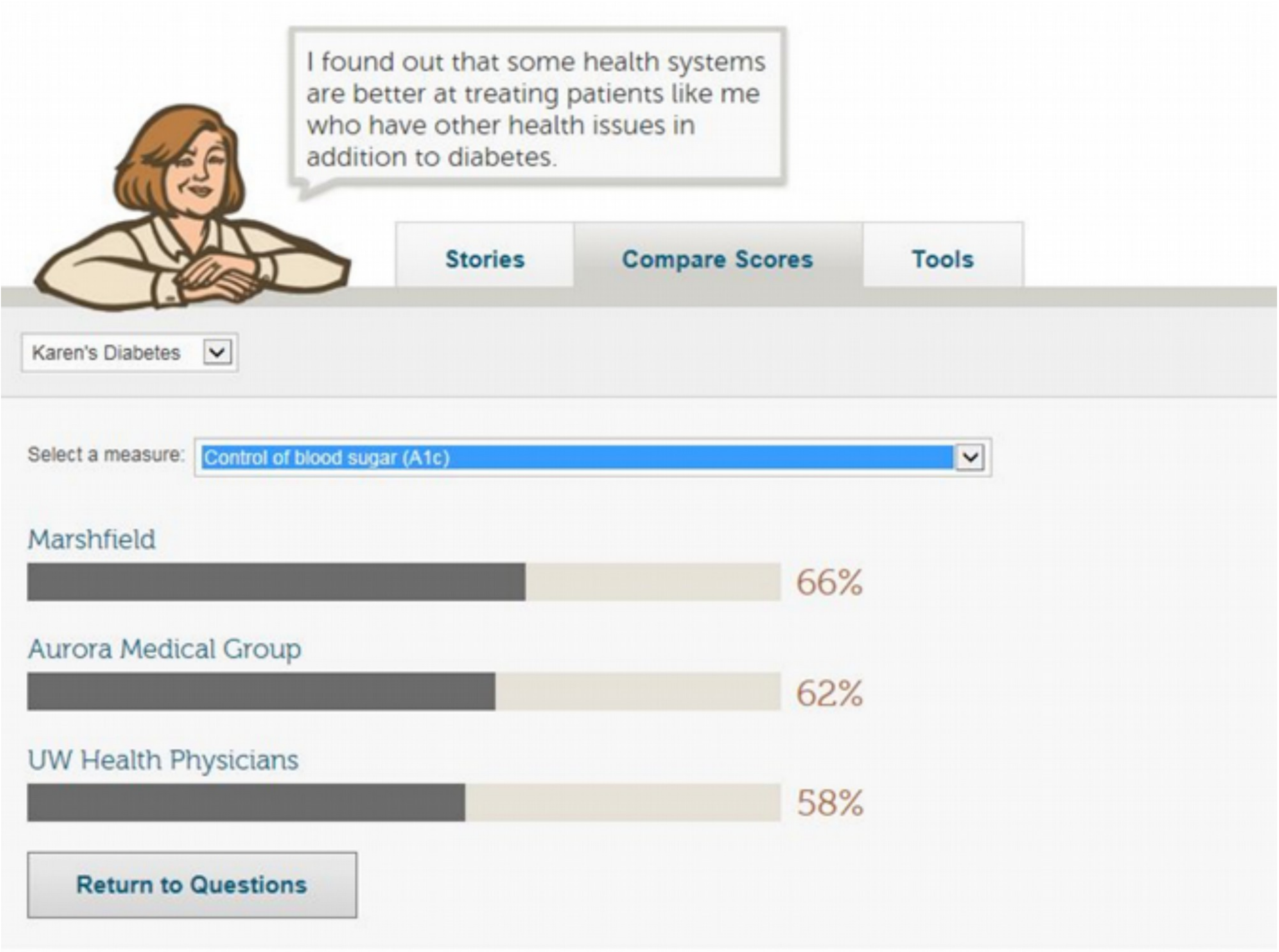

Supplement: Multimedia Appendix 1 [file jmir_v18i12e332_app1.pdf]
